# Calcium-Enriched Pumpkin Affects Serum Leptin Levels and Fat Content in a Rat Model of Postmenopausal Osteoporosis

**DOI:** 10.3390/nu13072334

**Published:** 2021-07-08

**Authors:** Natalia Wawrzyniak, Joanna Suliburska, Bartosz Kulczyński, Paweł Kołodziejski, Paweł Kurzawa, Anna Gramza-Michałowska

**Affiliations:** 1 Department of Human Nutrition and Dietetics, Faculty of Food Science and Nutrition, Poznań University of Life Sciences, Wojska Polskiego 31, 60-624 Poznań, Poland; natalia.wawrzyniak@up.poznan.pl (N.W.); joanna.suliburska@up.poznan.pl (J.S.); 2 Department of Gastronomy Science and Functional Foods, Faculty of Food Science and Nutrition, Poznań University of Life Sciences, Wojska Polskiego 31, 60-624 Poznań, Poland; bartosz.kulczynski@up.poznan.pl; 3 Department of Animal Physiology, Biochemistry and Biostructure, Faculty of Veterinary Medicine and Animal Science, Poznań University of Life Sciences, Wojska Polskiego 28, 60-637 Poznań, Poland; pawel.kolodziejski@up.poznan.pl; 4 Department of Clinical Pathology, Poznań University of Medical Sciences, Przybyszewskiego 49, 60-355 Poznań, Poland; pawel.kurzawa@skpp.edu.pl; 5 Department of Oncological Pathology, University Hospital of Lord’s Transfiguration, Partner of Karol Marcinkowski University of Medical Sciences, Szamarzewskiego 84, 60-569 Poznań, Poland

**Keywords:** postmenopausal osteoporosis, obesity, pumpkin, calcium, leptin

## Abstract

Because the world’s population is deficient in dietary calcium, it is important to search for new sources of this essential mineral for the bones and the entire body. One of the innovative foods that could act as such a source is pumpkin enriched with calcium lactate by means of osmotic dehydration. Providing the body with easily absorbable calcium may have beneficial effects on the reconstruction of bone tissue. Postmenopausal osteoporosis is associated with body weight and fat mass gain, and the aim of the present study was to evaluate the effect of consuming enriched pumpkin on the levels of adipokines and cytokines produced by the adipose tissue. This study was conducted on 12-month-old female Wistar rats that received nutritional intervention for 12 weeks. After termination of the rats, the levels of leptin, adiponectin, interleukin 31 and interleukin 33 in serum and adipose tissue were determined, and the femurs were examined histopathologically. It was demonstrated that calcium-enriched pumpkin reduced bone marrow femoral adipocytes and also markedly decreased serum leptin levels in groups of rats after ovariectomy, which was associated with a decrease of fat content. Additionally, it seems that calcium-enriched pumpkin may reduce body weight gain often observed after menopause.

## 1. Introduction

Osteoporosis is a disease of the skeletal system characterized by a reduction in bone mass and qualitative skeletal changes that cause an increase in bone fragility and higher fracture risk. The population group most at risk from osteoporosis is postmenopausal women due to decreased levels of estrogen, a hormone that protects bone in reproductive age from regulating bone turnover [1]. In 2010, it was reported that approximately 22 million women and 5.5 million men in Europe suffer from osteoporosis, and there have been 3.5 million bone fractures a year from the disease. The increasing incidence of osteoporotic fractures due to population aging leads to a reduction in quality of life, and therefore the treatment of this disease is essential from the perspective of public health [2]. Adequate supply of calcium (the main bone mineral) to the body is a preventive measure of osteoporosis. In this way, bone formation processes that increase bone mineral density are supported [3]. Unfortunately, the average calcium intake in the global population is far too low and often does not exceed even half of the requirement, which should be 1000 mg per day. For women over 50 years, it should be 1200 mg/day (with a upper limit of 2000 mg/day) [4].

Osteoblasts (bone tissue cells) and adipocytes (adipose tissue cells) are derived from the same precursor mesenchymal stem cells (MSCs). Although both tissues perform different functions in the body, their metabolisms interact with each other through adipokines (adipose tissue hormones) such as leptin and adiponectin. These factors not only have a paracrine effect on adipocytes, but they also cause endocrine disruption in distant tissues, including bones [5]. Postmenopausal bone mineral density is determined by body weight, among other factors, and excessive adipose tissue mass positively correlates with a low risk of osteoporosis, which indicates the preventive and therapeutic effect of adipokines against this disease. Adipose tissue also has another effect on bone—it expresses interleukin 33 (IL-33) [6], which, together with interleukin 31 (IL-31), regulates bone remodeling. One of the functions of IL-31 is to maintain the homeostasis of bone marrow cells responsible for the formation of osteoclasts (the cells that resorb bone). This interleukin also influences the intensity of inflammation, which increases the production of osteoclastogenic cytokines. The stimulation of IL-31 production occurs (among others) through IL-33, which in turn is released due to the stress of cell death. IL-33 is also responsible for the inhibition of osteoclast formation, which indicates the antiresorptive protective effect of bone tissue. Therefore, the IL-31/IL-33 axis influences the homeostasis of anabolic and catabolic bone processes, and their altered concentration may indicate the presence of osteoporosis [7].

Low calcium intake and the increased incidence of osteoporosis indicate the need to look for new solutions that could help to improve the bioavailability of calcium in the population groups most at risk of deficiency, and consequently also increase bone saturation with calcium, helping to maintain normal bone mineral density. One way to increase the amount of calcium in a meal is to use foods fortified with this mineral [8]. The present study used pumpkin as a plant matrix suitable for calcium enrichment, which also has many nutritional characteristics that support health, including hypoglycemic and hypolipemic effects [9]. The aim of this study was to determine the effect of pumpkin enriched with calcium lactate on changes in the level of adipokines and cytokines, which are among the indicators of the occurrence of osteoporosis.

## 2. Materials and Methods

### 2.1. Materials and Reagents

To enrich pumpkins from domestic organic farming, it was necessary to purchase calcium lactate and inulin. Adipose tissue homogenization was performed with PBS (phosphate-buffered saline). The concentration of the examined parameters in the tissues was assessed with the use of enzyme-linked immunoassay (ELISA) kits for rat leptin and adiponectin, which were purchased from Mediagnost (Reutlingen, Germany), and rat interleukin IL-31 and IL-33, which were purchased from Shanghai Sunred Biological Technology Co. (Shanghai, China).

### 2.2. Animals

Female 12-week-old Wistar rats were purchased from the Wielkopolska Centre of Advanced Technologies of Adam Mickiewicz University (Poznań, Poland). The animals were maintained under standard conditions: individually in cages, 12 h light/dark cycle, allowed to acclimate for 1 week. The animal experiment was performed in accordance with the guidelines for the care and use of laboratory animals.

### 2.3. Experimental Protocols

The study was conducted on 50 rats. All rats were fed an AIN-93M diet [10]. The animals were randomly divided into 5 groups, with 10 animals in each group. At the beginning of the experiment, the total body weight of the rats did not differ between the groups. Each rat was in a separate cage, which was positioned so that the rats could see each other, reducing the stress associated with the study. At the beginning of the experiment, 40 rats were ovariectomized (OVX) to establish the postmenopausal osteoporosis rat model.

After 7 days of convalescence, the nutritional intervention was initiated for 12 weeks. The control group (C) and one of the ovariectomized groups (OVX) were given the standard diet (without modification), the OVX-D group was fed a diet deprived of calcium, the OVX-CL group was fed a diet with calcium lactate, the OVX-PCL group was fed with calcium lactate-enriched pumpkin. Each diet, except for the calcium-deprived diet, contained 5 g of calcium per 1 kg of the diet. A calcium-deprived diet was achieved by omitting the addition of calcium carbonate in the standard diet. Calcium carbonate is a source of calcium in the AIN-93M diet. The pumpkin was enriched with calcium lactate in the process of osmotic dehydration with the use of an osmotically active substance (inulin). The pumpkin characteristics and composition were shown in our previous study [9]. One gram of lyophilizate-enriched pumpkin with calcium lactate contains 28 mg of calcium. Pumpkin enriched with calcium lactate was added at the rate of 180 g per 1 kg of the OVX-PCL deprived diet, while 27.22 g of calcium lactate per 1 kg was added to the OVX-CL deprived diet. The animals were allowed to eat the diet and drink deionized water ad libitum throughout the experiment. The rats in each group were weighed weekly and food consumption was recorded daily. After the end of the experiment, body composition analysis of all animals was performed in Bruker’s LF90II Body Composition Analyzer. The rats in each group were then decapitated, and blood and perioral adipose tissue samples were collected and stored at −80 °C The animals were included in the study if they underwent successful ovariectomy.

### 2.4. Calcium Analysis in Diets

The 1 g samples of diets were ashed in a muffle furnace at 450 °C until complete mineralization and then dissolved in 1 mol/L nitric acid (Merck, Kenilworth, NJ, USA). The mineral content of the samples was determined by flame atomic absorption spectrometry (AAS-3, Zeiss spectrometer), after appropriate dilution with deionized water and LaCl_3_ (0.5%). The methods were validated by a simultaneous analysis of the reference material (Brown Bread BCR191, Sigma-Aldrich, St. Louis, MO, USA), with an accuracy of 92%. The calcium contents in the diets are presented in Table 1.

### 2.5. Serum and Adipose Tissue Parameters

Blood was centrifuged at 1200× *g* for 10 min at 4 °C and adipose tissue was homogenized in PBS (phosphate-buffered saline) by centrifugation at 7000× *g* for 20 min. Leptin, adiponectin, IL-31, and IL-33 concentrations were measured in serum and homogenized adipose tissue with ELISA kits (*n* = 10 in each experimental group).

### 2.6. Histopathological Analysis

The resected specimens, in the form of femoral bones, were fixed in 10% buffered formalin for 24 h. Next, they were decalcified in the decalcifying solution (Osteodec bone marrow biopsy decalcifying solution) for another 3 h. After that, each of the bone specimens were routinely processed and embedded in paraffin blocks separately. Two-micrometer tissue sections were cut from the paraffin blocks and stained with hematoxylin and eosin (H&E); three sections were cut from each tissue sample. Each slide contained two femoral bone sections with the content of bone marrow. The bone marrow of each bone was analyzed under a light microscope (Leica, Allendale, NJ, USA) and the ratio of adipose tissue to bone marrow was assessed on a high-power field (400× magnification) by two separate scientists. The ratio of adipose tissue was virtually assessed on a light microscope in 5 different high-power field areas (400×; area of 0.25 mm^2^). The number of osteoblasts were counted per 1 HPF (high-power field—400× magnification, or area of 0.25 mm^2^).

### 2.7. Statistical Analysis

All results are presented as means ± standard deviation (SD). The Shapiro–Wilk test was performed to check the variable distributions for normality. The Pearson correlation coefficient was used to assess the correlation between parameters. Statistical analyses were performed using Statistica (StatSoft, Tulsa, OK, USA). Statistical differences were assessed using one-way ANOVA, followed by Tukey’s post hoc test for normal distribution of values, and Kruskal–Wallis test and multiple comparisons with rank sum for non-normal data (*p* < 0.05).

## 3. Results

**Figure 1:**
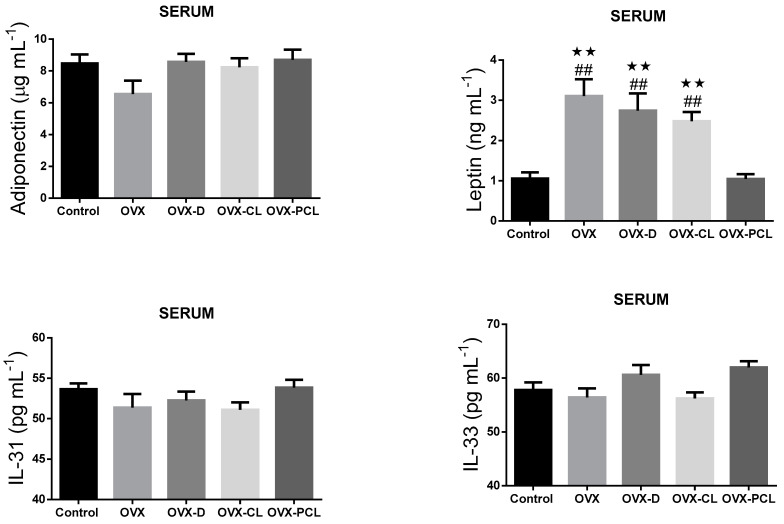
The levels of adipokines and cytokines in the serum of rats. OVX: ovariectomized group; OVX-D: ovariectomized group with a deprivation of calcium; OVX-CL: ovariectomized group with a deprivation of calcium with the addition of calcium lactate; OVX-PCL: ovariectomized group with a deprivation of calcium with the addition of pumpkin enriched with calcium lactate. IL-31: interleukin 31; IL-33: interleukin 33; statistically significant changes are marked ** *p* < 0.01 compared to control and ## *p* < 0.01 compared to OVX-PCL.

**Figure 2:**
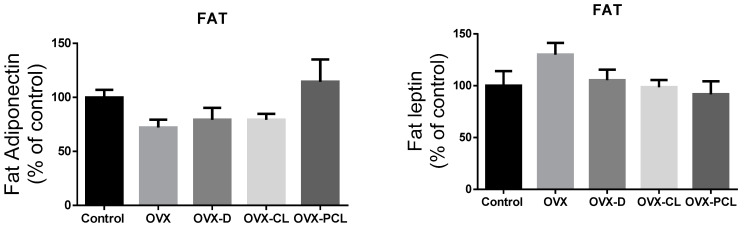

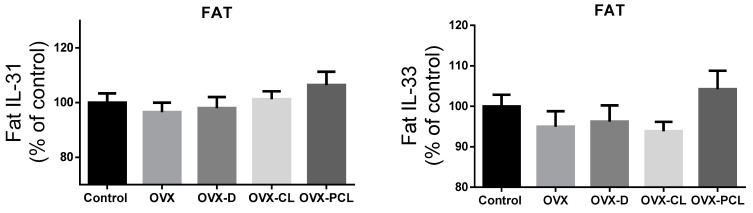
The levels of adipokines and cytokines in the adipose tissue of rats. OVX: ovariectomized group; OVX-D: ovariectomized group with a deprivation of calcium; OVX-CL: ovariectomized group with a deprivation of calcium with the addition of calcium lactate; OVX-PCL: ovariectomized group with a deprivation of calcium with the addition of pumpkin enriched with calcium lactate. IL-31: interleukin 31; IL-33: interleukin 33.

**Figure 3:**
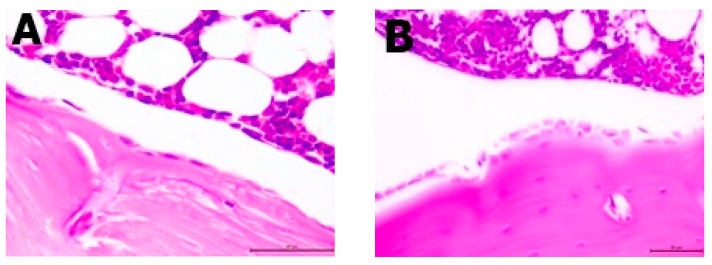
Differences between the number of osteoblasts. (**A**) Few osteoblasts along the bones of the representative of the OVX group (H&E; 10×); (**B**) numerous clusters of osteoblasts arranged along the bones of the representative of the OVX-D group (H&E; 40×).

**Figure 4:**
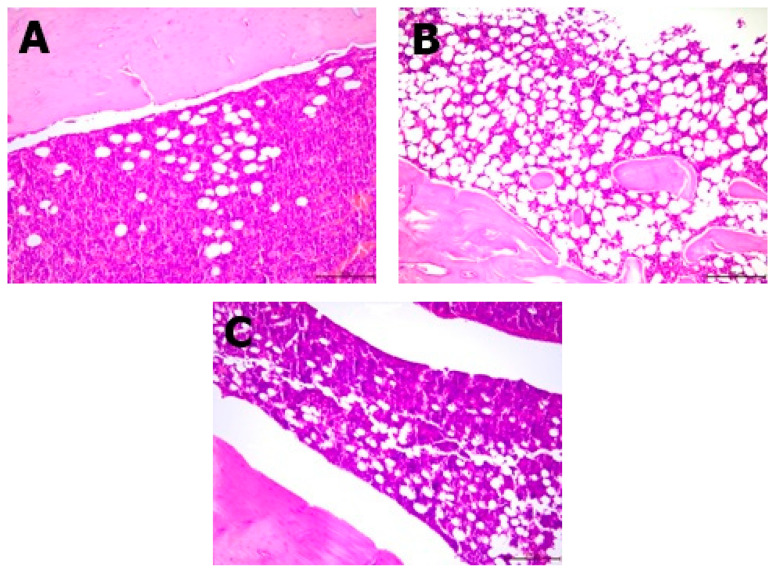
Differences between the amount of bone marrow femoral adipocytes. (**A**) Minor bone marrow femoral adipocytes in a representative from the control group (H&E; 10×); (**B**) severe bone marrow femoral adipocytes in a representative from the OVX group (H&E; 20×); (**C**) minimized bone marrow femoral adipocytes in a representative of the enriched pumpkin-fed group (OVX-PCL; H&E; 10×).

The results of the study are presented in Table 1, Table 2, Table 3 and Table 4 and Figure 1, Figure 2, Figure 3 and Figure 4. Table 1 presents the calcium content of the diets. The lowest calcium content was recorded in OVX-D, which differed statistically from other diets with the correct calcium content.

Daily calcium intake varied significantly between the groups. The OVX-D group received a negligible amount of calcium compared to the others (Table 2). Body weight and fat content in the ovariectomized groups (OVX, OVX-D, and OVX-CL) were significantly higher than in the control group. It was observed that the content of fat tissue in groups OVX, OVX-D, and OVX-CL was almost twice as high as in group C. Groups with calcium-enriched pumpkin had body weight and fat mass comparable to the control group.

It was found that the levels of serum leptin concentration in the OVX, OVX-D, and OVX-CL groups were significantly higher than in the control and OVX-PCL groups (Figure 1). Leptin concentration in the C group and OVX-PCL group was almost three times lower than in the three other groups (OVX, OVX-D, OVX-CL). There were no marked differences in the levels of other parameters: adiponectin, IL-31 and IL-33 in adipose tissue and serum, and leptin in adipose tissue (Figure 1 and Figure 2).

Histopathological analysis showed changes between the groups in the bone structure (Table 3, Figure 3 and Figure 4). An increased amount of bone marrow femoral adipocytes was observed in the ovariectomized rats. The enriched pumpkin seemed to reduce the effect of ovariectomy on the bone marrow. A calcium-deprived diet significantly increased the number of osteoblasts in femurs in comparison to control and OVX groups.

A significant positive correlation between serum leptin concentration and body weight and fat tissue content was observed (Table 4). It was found that there was a negative correlation between serum leptin level and adiponectin concentration in adipose tissue. There was also a positive correlation between leptin concentration and bone marrow femoral adipocytes.

## 4. Discussion

In the present study, significant increases in body weight and body fat in ovariectomized rats were observed. The exception was a group of rats fed with pumpkin enriched with calcium lactate—the body composition of these animals and their serum leptin levels did not differ from the control group. Postmenopausal women experience increased body weight and the accumulation of abdominal fat, as well as decreased subcutaneous fat due to decreased estrogen levels [11]. Both estrogen and leptin receptors are found in the same neurons in the ventromedial hypothalamus, arcuate nucleus, or the preoptic area which coordinate both gonadal function and metabolism. Thus, estrogen is responsible not only for the regulation of reproduction, but also influences the maintenance of normal body weight; meanwhile, leptin, in addition to regulating appetite, also participates in modulating neuroendocrine functions of reproduction [12]. After menopause, leptin levels fluctuate with changes in bodyweight. Hyperleptinemia in obese women results in increased bone mass density because this fat tissue hormone stimulates osteoblastogenesis and inhibits the formation of osteoclasts, and affects the production of collagen and bone mineralization. On the other hand, in postmenopausal women with normal body weight, serum leptin levels are much lower [13]. Although leptin has a beneficial effect on bone, its elevated levels—and thus also the increased content of visceral fat—can lead to undesirable consequences. A relationship has been observed between increased concentration of leptin and the symptoms of the metabolic syndrome, including atherosclerosis, insulin resistance, and high blood pressure, which may result in cardiovascular disease [14]. Increased serum leptin levels can lead to inflammation, as this adipokine is involved in the production of pro-inflammatory cytokines. Increased inflammation may result in the formation of breast cancer, which also confirms the ability of leptin to promote angiogenesis in neoplastic cells [15].

In the group of rats that underwent ovariectomy, it is clearly visible that the serum leptin level, body weight, and adipose tissue content were significantly higher than in the control group, which confirms the positive correlation between leptin concentration and body weight after menopause. The group of rats fed with calcium-enriched pumpkin, as the only intervention group, did not differ statistically from the control group in terms of serum leptin concentration, body weight, or adipose tissue content, which indicates a beneficial effect of enriched pumpkin on energy homeostasis in estrogen deficiency. A possible mechanism that could explain these changes is the effect of the bioactive compounds of pumpkin on body weight and adipose tissue. One of the groups of plant compounds conducive to weight loss are antioxidants. After menopause, estrogen deficiency increases reactive oxygen species (ROS), whereby osteoblastogenesis is impaired and bone resorption is increased, leading to a decrease in bone mineral density [16]. Increased reactive oxygen species also leads to cell dysfunction, altered signaling and transport between cells, and decreased energy metabolism, which can lead to weight gain [17]. Pumpkin has high antioxidant potential, which was studied in our previous research [18]. Antioxidants can reverse the negative effects of estrogen deficiency by decreasing oxidative stress, which destroys bone tissue and increases body weight. One of the antioxidants contained in pumpkin is β-cryptoxanthin, which is a carotenoid that can reduce the oxidative stress caused by menopause, and thus also reduce body weight and increase anabolic processes of bone formation. β-Cryptoxanthin is absorbed much better (over seven times better) than alpha- or beta-carotene—carotenoids that are also found in pumpkin [19]. Pumpkin also contains other antioxidants that reduce oxidative stress, such as zeaxanthin, lutein, lycopene from the carotenoid group, and rutin and kaempferol from the flavonol group [9,19]. 

The group consuming the diet with the addition of enriched pumpkin was characterized by the lowest concentration of leptin and amount of adipocytes in femurs among ovariectomized rats. Leptin has been shown to regulate bone mass by enhancing the sympathetic nervous system in the hypothalamus by leptin receptors, and it interferes with bone formation through adrenergic receptors in osteoblasts. Sympathetic signaling is very important in the pathogenesis of osteoporosis, as it has been proven that mice without this signaling will not develop osteoporosis due to ovarian failure. Leptin also increases bone resorption by increasing the expression of RANKL in osteogenic cells, thereby stimulating the activity of osteoclasts [20]. Bone marrow fat is also involved in the pathogenesis of osteoporosis, and by increasing the release of adipokines, bone resorption is enhanced [21]. The increase in adipogenesis and decrease in bone osteogenesis as a result of elevated serum leptin levels occurs through bone mesenchymal stromal cells, which are involved in the signaling of leptin to its receptors. Skeletal stem cells determine the expression of leptin receptors, without which the signaling of leptin by the receptors would not be possible, and an increased concentration of leptin would not lead to an increase in the number of fat cells in the bone marrow and a decrease in bone formation. This demonstrates the role of increased leptin levels in the adipocyte content in the bone marrow [22]. This fact explains the observation of the highest percentage of bone marrow femoral adipocytes in rats with the highest serum leptin concentration (OVX-D group).

As already mentioned, leptin leads to an increase in osteoblastogenesis, which would confirm the highest number of osteoblasts in the group with high leptin concentration (OXD-D groups) [13]. Nevertheless, the OVX group had a similar number of osteoblasts as the control group, and the concentration of leptin was the highest among the studied groups. The reason for the large number of osteoblasts should therefore be sought in the mechanisms associated with insufficient calcium intake in the diet. Osteoblasts are derived from mesenchymal stem cells and are located along the length of the bone [23]. Osteoblasts are involved in bone formation by synthesizing new bone matrix, the structure of which is maintained by osteocytes. Insufficient supply of calcium contributes to the deterioration of bone mineralization and bone formation, and thus the number of osteoblasts is also reduced [24]. This was not confirmed by the results of this experiment, but a diet low in calcium may lead to many other mechanisms. Low levels of calcium in the blood increase the concentration of parathyroid hormone, which mobilizes calcium from the bones and increases its absorption. A diet low in calcium and long-term elevated levels of parathyroid hormone lead to two conflicting mechanisms: an increase in the number of osteoblasts and bone formation as well as resorption stimulation [25].

There was also a negative correlation between serum leptin concentration and adiponectin level in adipose tissue. Adipose tissue secretes both of these adipokines, of which leptin is atherogenic and pro-inflammatory, while adiponectin has a protective effect. Abdominal obesity in patients is associated with higher levels of leptin and lower levels of adiponectin, which confirms their negative correlation [26]. During the increase in body weight, leptin stimulates the secretion of adiponectin, so their correlation is positive. However, in a later stage, this signaling is impaired due to the increased secretion of caveolin-1 (Cav1), so leptin remains high in obese individuals, and the adiponectin level is not increased [27].

No effect of nutritional intervention was observed in rats on the changes in adiponectin, leptin, interleukin-31, and interleukin-33 in adipose tissue; or in adiponectin, interleukin-31, and interleukin-33 in the serum. Similarly, no effect of calcium supplementation on plasma adiponectin levels was reported in obese women in [28]. However, in ovariectomized rats, the effect of estrogen reduction on changes in adiponectin levels was also not observed in [29].

### Limitations

There are several limitations in this study that may have affected its results. No sham group was included in the study, and therefore no effect of sham surgery on rats was noted. The focus was on the determination of leptin, adiponectin, interleukin-31 and interleukin-33 in serum and adipose tissue. However, other adipokine markers and cytokines would provide a more comprehensive picture of the effect of fortified pumpkin on osteoporosis rates. The indices of oxidative stress status were not measured and antioxidant parameters were not compared between the groups. In this study, we did not involve bone metabolism parameters, which could broaden interpretation and discussion of the results.

## 5. Conclusions

Pumpkin enriched with calcium lactate decreased serum leptin levels, and reduced body fat and body weight in ovariectomized rats. It seems that calcium-enriched pumpkin may reduce the body weight gain often observed after menopause. Further clinical studies are needed to confirm this beneficial effect of calcium-enriched pumpkin in postmenopausal women and to explain the mechanism related to the obtained results.

## Figures and Tables

**Table 1:** Calcium content in diets (mean and standard deviation).

Group	Control and OVX	OVX-D	OVX-CL	OVX-PCL
Calcium content (mg/g)	5.63 ± 0.37 ^b^	0.64 ± 0.04 ^a^	5.68 ± 0.24 ^b^	5.77 ± 0.15 ^b^

OVX: ovariectomized group; OVX-D: ovariectomized group with a deprivation of calcium; OVX-CL: ovariectomized group with a deprivation of calcium with the addition of calcium lactate; OVX-PCL: ovariectomized group with a deprivation of calcium with the addition of pumpkin enriched with calcium lactate. ^a,b^: significant differences between groups (*p* < 0.05)

**Table 2:** Daily intake of the diet, final body weight, and fat content in rats (mean and standard deviation).

Group	Control	OVX	OVX-D	OVX-CL	OVX-PCL
Daily food intake (g)	25.08 ± 0.63	25.11 ± 1.70	26.14 ± 1.87	25.90 ± 0.55	24.31 ± 1.26
Daily calcium intake (mg)	141.12 ± 3.56 ^b^	141.3 ± 9.57 ^b^	16.77 ± 1.2 ^a^	147.03 ± 3.11 ^b^	140.31 ± 7.26 ^b^
Final body weight (g)	325.86 ± 25.97 ^a^	421.90 ± 55.10 ^b^	441.00 ± 70.97 ^b^	428.40 ± 51.10 ^b^	384.11 ± 34.02 ^a,b^
Fat tissue (g)	122.08 ± 35.78 ^a^	238.66 ± 55.07 ^b,c^	246.19 ± 83.14 ^c^	252.27 ± 47.47 ^c^	167.79 ± 31.34 ^a,b^
Fat tissue (%)	36.95 ± 8.63 ^a^	54.43 ± 10.26 ^b,c^	55.73 ± 11.11 ^c^	58.59 ± 5.89 ^c^	43.06 ± 7.90 ^a,b^

OVX: ovariectomized group; OVX-D: ovariectomized group with a deprivation of calcium; OVX-CL: ovariectomized group with a deprivation of calcium with the addition of calcium lactate; OVX-PCL: ovariectomized group with a deprivation of calcium with the addition of pumpkin enriched with calcium lactate. ^a–c^: significant differences between groups (*p* < 0.05).

**Table 3:** Percentage of bone marrow femoral adipocytes and the number of osteoblasts (mean and standard deviation).

Group	Control	OVX	OVX-D	OVX-CL	OVX-PCL
Bone marrow femoral adipocytes (%)	8.5 ± 3.4 ^a^	43.0 ± 10.6 ^c^	40.9 ± 8.3 ^c^	43.0 ± 8.2 ^c^	30.0 ± 7.1 ^b^
Number of osteoblasts	9.9 ± 3.4 ^a^	9.0 ± 5.5 ^a^	22.8 ± 15.1 ^b^	16.6 ± 7.8 ^a,b^	18.0 ± 3.6 ^a,b^

OVX: ovariectomized group; OVX-D: ovariectomized group with a deprivation of calcium; OVX-CL: ovariectomized group with a deprivation of calcium with the addition of calcium lactate; OVX-PCL: ovariectomized group with a deprivation of calcium with the addition of pumpkin enriched with calcium lactate. ^a–c^: significant differences between groups (*p* < 0.05).

**Table 4:** Significant correlations (Pearson correlation coefficient, r) between leptin and other analyzed parameters in rats.

Parameter	Body Weight (g)	Fat Tissue (g)	Fat Tissue (%)	ADI_AT (% of Control)	Bone Marrow Femoral Adipocytes (%)
LEP_S (ng/mL)	0.75	0.88	0.83	−0.49	0.49

LEP_S: serum leptin concentration; ADI_AT: adipose tissue adiponectin concentration (*p* < 0.05).

## Data Availability

The data used to support the findings of this study can be made available by the corresponding author upon request.
